# Positive Effects of PI3K/Akt Signaling Inhibition on PTEN and P53 in Prevention of Acute Lymphoblastic Leukemia Tumor Cells

**DOI:** 10.15171/apb.2019.056

**Published:** 2019-08-01

**Authors:** Elahe Naderali, Behnaz Valipour, Amir Afshin Khaki, Jafar Soleymani Rad, Alireza Alihemmati, Mohammad Rahmati, Hojjatollah Nozad Charoudeh

**Affiliations:** ^1^Stem Cell Research Center, Tabriz University of Medical Sciences, Tabriz, Iran.; ^2^Department of Anatomical Sciences, Faculty of Medicine, Tabriz university of Medical Sciences, Tabriz, Iran.; ^3^Department of Clinical Biochemistry Sciences, Faculty of Medicine, Tabriz University of Medical Sciences, Tabriz, Iran.

**Keywords:** PI3K/Akt signaling, MK-2206, BKM-120, ALL, PTEN, P53

## Abstract

***Purpose:*** The PI3K/Akt signaling pathway regulates cell growth, proliferation and viability in hematopoietic cells. This pathway always dysregulates in acute lymphoblastic leukemia (ALL). PTEN and P53 are tumor suppressor genes correlated with PI3K/Akt signaling pathway, and both have a tight link in regulation of cell proliferation and cell death. In this study, we investigated the effects of dual targeting of PI3K/Akt pathway by combined inhibition with nvp-BKM-120 (PI3K inhibitor) and MK-2206 (Akt inhibitor) in relation with PTEN and P53 on apoptosis and proliferation of leukemia cells.

***Methods:*** Both T and B ALL cell lines were treated with both inhibitors alone or in combination with each other, and induction of apoptosis and inhibition of proliferation were evaluated by flow cytometry. Expression levels of PTEN as well as p53 mRNA and protein were measured by real-time qRT-PCR and western blot, respectively.

***Results:*** We indicated that both inhibitors (BKM-120 and MK-2206) decreased cell viability and increased cytotoxicity in leukemia cells. Reduction in Akt phosphorylation increased PTEN and p53 mRNA and p53 protein level (in PTEN positive versus PTEN negative cell lines). Additionally, both inhibitors, particularly in combination with each other, increased apoptosis (evaluated with Annexin V and caspase 3) and reduced proliferation (Ki67 expression) in leukemia cells. However, administration of IL7 downregulated PTEN and P53 mRNA expression and rescued cancer cells following inhibition of BKM-120 and MK-2206.

***Conclusion:*** This investigation suggested that inhibition of Akt and PI3K could be helpful in leukemia treatment.

## Introduction

Acute lymphoblastic leukemia (ALL) is the most common and aggressive form of cancer in children.^1,2^ Despite significant improvements in the therapeutic outcomes and high cure rates of childhood ALL rather than adults,^3,4^ relapsed ALL is a leading cause of death in children and adults.^5-7^ ALL is a malignant neoplasm of early lymphoid precursor cells commonly affecting B cell lineage.^8,9^ The PI3K/Akt signaling pathway plays a critical role in regulating many cellular and biological functions such as proliferation, survival, apoptosis, cell cycle progression, protein synthesis and differentiation.^10,11^ Phosphatidylinositol (4,5)-bisphosphate (PIP2) phosphorylates to phosphatidylinositol (3,4,5)-trisphosphate (PIP3) via activated phosphatidylinositol 3 kinase (PI3K) in the plasma membrane.^12^ PIP3 binds to pleckstrin homology domains and facilitates the activation of several downstream effectors, particularly Akt.^13,14^ Akt plays an important role in the activation and phosphorylation of mammalian target of rapamycin (mTOR).^15,16^ PTEN as a tumor suppressor antagonizes PI3K/Akt pathway by dephosphorylation of PIP3 to PIP2.^17^ Dysregulation of the PI3K/Akt pathway is one of the most frequent events in both B-cell and T-cell ALL, causing to enhance proliferation, survival, and drug resistance.^18,19^ P53 is another tumor suppressor with a short lifespan and a non-abundant nuclear protein in normal cells.^20,21^ It is activated in response of cell stresses and upregulates target genes for cell cycle arrest, growth inhibition and apoptosis.^22^ PTEN and p53 are tightly linked together. Both of them regulate cell proliferation and cell death. Their activity is dependent on each other.^23^ Conventional cancer therapies, including chemotherapy and radiotherapy, could not completely prevail overexpression and activation of the PI3K/Akt pathway in cancer cells.^24,25^ To overcome drug resistance and side effects of traditional cancer therapies, application of various small molecule inhibitors could increase the specificity and efficacy of cancer targeting. Numerous preclinical studies have been focused mainly on PI3K/Akt signaling as an attractive object for targeted therapy in leukemia.^26^

BKM120 (NVP-BKM120 or Buparlisib) is an orally bioavailable, highly potent and selective pan-class I PI3K inhibitor preventing the binding of ATP to PI3K active sites. It has exhibited strong antiproliferative activities, antitumor effects and has induced apoptosis in various cancers.^27^ In addition, MK-2206 is an orally active, selective and potent allosteric inhibitor of Akt; preclinical studies have demonstrated its efficacy in the treatment of various cancers.^28^ Here, we evaluated whether inhibition of PI3K/Akt signaling by BKM-120 and MK-2206 would affect the phosphorylation and activation of Akt in correlation with PTEN and P53. Finally, we used interleukin-7 (IL-7), as a growth factor for the development of T and pre B-ALL, to understand whether IL-7 would rescue PI3K/AKT signaling inhibition.

## Materials and Methods

### Cell lines and cell cultures

The B-ALL (Nalm-6 and Reh-6)^29^ and T-ALL (Dnd-41^30^ and Molt-4^31^ ) cell lines were purchased from the National Cell Bank of Iran (Pasteur Institute, Tehran, Iran). Nalm-6, Reh-6 and Molt-4 cell lines are PTEN positive whereas Dnd-41 cell line is PTEN negative. Four ALL cell lines were cultured in RPMI 1640 medium supplemented with 10% heat-inactivated FBS (Gibco), 100 mg/mL streptomycin and 100 units/mL penicillin G (Sigma, St Louis, MO, USA) in a 37°C incubator with a humidified atmosphere of 5% CO2.

### MTT assays

A total of 200 µL cell suspensions were seeded and treated in triplicate in flat bottomed 96-well plates (3 × 10^4^cells per well) with a different dose of BKM-120 and MK-2206 inhibitors as incubation at 37°C with 5% CO2. Untreated cells served as control. Following treatment for 24, 48 and 72 hours with BKM-120 and MK-2206 inhibitors, colorimetric methylthiazol tetrazolium bromide (MTT) solution was added to a final concentration of 5 mg/mL in each well and incubation was prolonged for 4 hours at 37°C. After, acidified detergent (10% SDS in 0.01 M HCl) was added to each well and incubated overnight at 37°C with 5% CO2 due to solubilization of the formazan. Complete solubilization of the purple formazan crystals by SDS was measured by ELISA reader. We used GraphPad Prism software to determine IC50 value of inhibitors, using regression analysis to fit a dose- response curve.

### RNA preparation, cDNA synthesis, quantitative RT-PCR and RT-PCR

The total RNA was extracted from 1×10^6^ cells using YTA kit (Yekta Tajhiz Azma, Iran) according to the silica based method. Then First-strand cDNA was synthesized via reverse transcription of 1 μg of total RNA by Prime Script^TM^ RT reagent kit (Takara, Japan) according to the manufacturer’s instructions. The cDNA was amplified using SYBR Green (Prime Script RT Master Mix, Takara, Tokyo, Japan). Real-time PCR was performed using the Corbett RotorGene™ 6000 HRM (Corbett Research, Australia) thermocycler. PCR primers were designed using Oligo7 software.^32^ Primer pairs sequence (5′3′) used for PCR, including Akt (forward: ATTGTGAAGGAGGGTTGGCTG and reverse: CTTGAGGAGGAAGTAGCGTGG), PTEN (forward: AGGAAGTGAATCTGTATTGGG and reverse: TTGCTGTGTTTCTTACCTATG), P53 (forward: TCAGTCTACCTCCCGCCATAA and reverse: AGTGGGGAACAAGAAGTGGAG), GAPDH (forward: CAAGATCATCACCAATGCCT and reverse: CCCATCACGCCACAGTTTCC).

### Antibodies and flow cytometry analysis

To assess cell proliferation, flow cytometric analysis was performed using PE-conjugated monoclonal anti-Ki-67 antibody (clone: 20Raj1, Bioscience) and apoptosis performed by Caspase 3 kit (BD Biosciences, San Diego, CA). For intracellular staining, cells were collected and washed twice with ice-cold PBS, resuspended with BD cytofix/cytoperm solution and incubated at 4°C for 20 minutes, cells were washed twice using Perm**/**Wash buffer and incubated with anti-Ki-67 or anti-caspase 3 antibody at room temperature for 20 minutes and analyzed by BD FACS Calibur with FlowJo (7.6.1) software for acquisition and analysis.

Also, flow cytometric analysis was performed using Annexin V-FITC/PI staining. Following 48h treatment, cells were collected and rinsed twice with ice-cold PBS. Then cells washed and incubated with Annexin V-FITC/PI (eBiosciences) for 15 min at room temperature. Cells were analyzed by BD FACS Calibur with FlowJo (7.6.1) software for acquisition and analysis.

### Western blot analysis

Cell lines were treated with inhibitors for 48 hours and lysed in RIPA buffer (Santa Cruz Biotechnology, Santa Cruz, CA, USA) supplemented with the protease and phosphatase inhibitor cocktail. Protein concentration was determined using the BCA Protein Assay kit (Pierce, Rockford, IL, USA). The lysates were diluted with sample loading buffer and equal amounts of protein extracts were separated on 12% SDS–PAGE gels and transferred to polyvinylidene difluoride membranes (GE Healthcare, Amersham, Buckinghamshire, UK). The membranes were blocked for 1 hour at room temperature with 5% low-fat dry milk or BSA. The membrane was incubated with primary monoclonal antibodies β-actin (1:1000), Akt, p-Akt, PTEN, p-PTEN and p53 (1:500, Cell Signaling Technologies) for overnight at 4˚C. After washing for three 10-minute, the membrane was incubated with a horseradish peroxidase-labeled goat anti-rabbit secondary antibody for 1 hour. Then, the blots were visualized using a chemiluminescent detection Kit (Roche, 11520709001) and X-ray film (Fujifilm, Sheffield, UK).

## Results

### Inhibition of PI3K/Akt signaling have cytotoxic effects on ALL cell lines

The anti-proliferative effects of BKM120 and MK-2206 in leukemia cell lines were examined in T-ALL (Molt-4 and Dnd-41) and B-ALL (Nalm-6 and Reh-6) cell lines with distinct concentrations of BKM120 and MK-2206 (between 0.25 and 10 μM) for 24, 48 and 72 hours.

Both BKM120 and MK-2206 reduced cell viability on Nalm-6, Reh-6 and Molt-4 cell lines in a concentration**-**dependent manner, whereas Dnd-41 cell line showed a much lower sensitivity. After 24, 48 and 72 hours of treatment with inhibitors, the IC50 of each cell line was measured. The best IC50 was achieved after 48 hours of treatment. Thus, BKM-120 IC50 of Nalm-6 (9.15 μM), Reh-6 (10.15 μM) Molt-4 (8.61 μM) and Dnd-41 (37.01 μM) cell lines and MK-2206 IC50 of Nalm-6 (0.79 μM), Reh-6 (0.89 μM) Molt-4 (2.22 μM) and Dnd-41 (24.25 μM) cell lines was defined.

**Figure 1:**
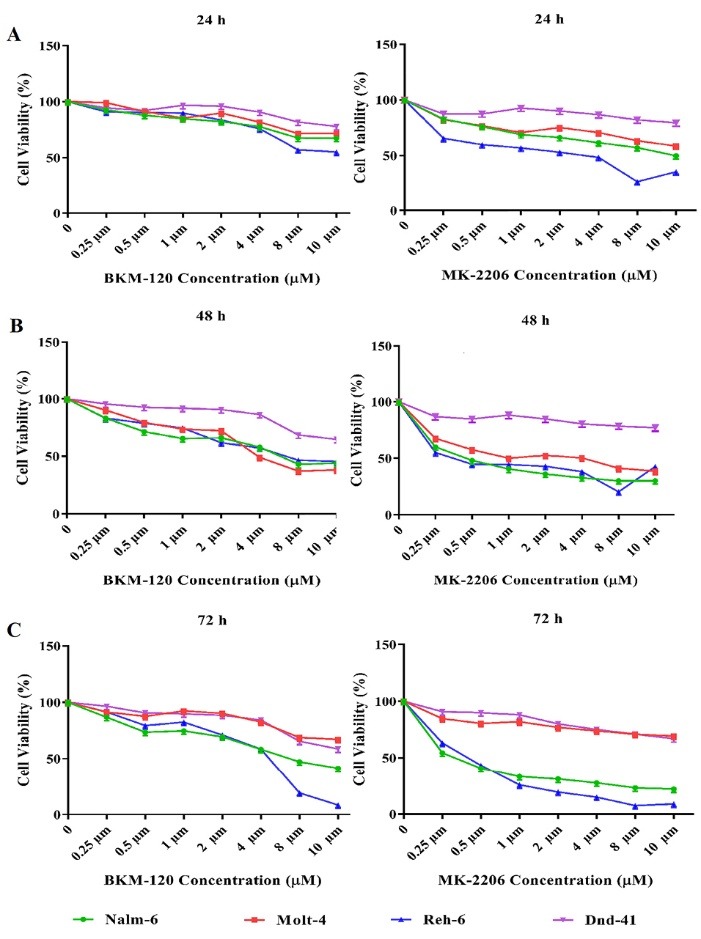
**Effect of BKM-120 and MK-2206 on T and B-ALL cell lines viability**. Nalm-6, Reh-6, Dnd-41 and Molt-4 cell lines were treated with indicating concentrations of inhibitors for (**A)** 24 h (**B),** 48 h and (**C)** 72 h. Results are the mean of three different experiments *P* < 0.0001.

Dnd-41 as a PTEN positive T cell line showed a much lower sensitivity to inhibitors in comparison to Molt-4 as a PTEN negative T cell line, but PTEN positive pre-B cell lines, Nalm-6 and Reh-6, similar to Molt-4 displayed the cytotoxic effect in response to inhibitors (Figure 1).

### Inhibition of PI3K/Akt signaling induced apoptosis and reduced proliferation in ALL cell lines

**Figure 2:**
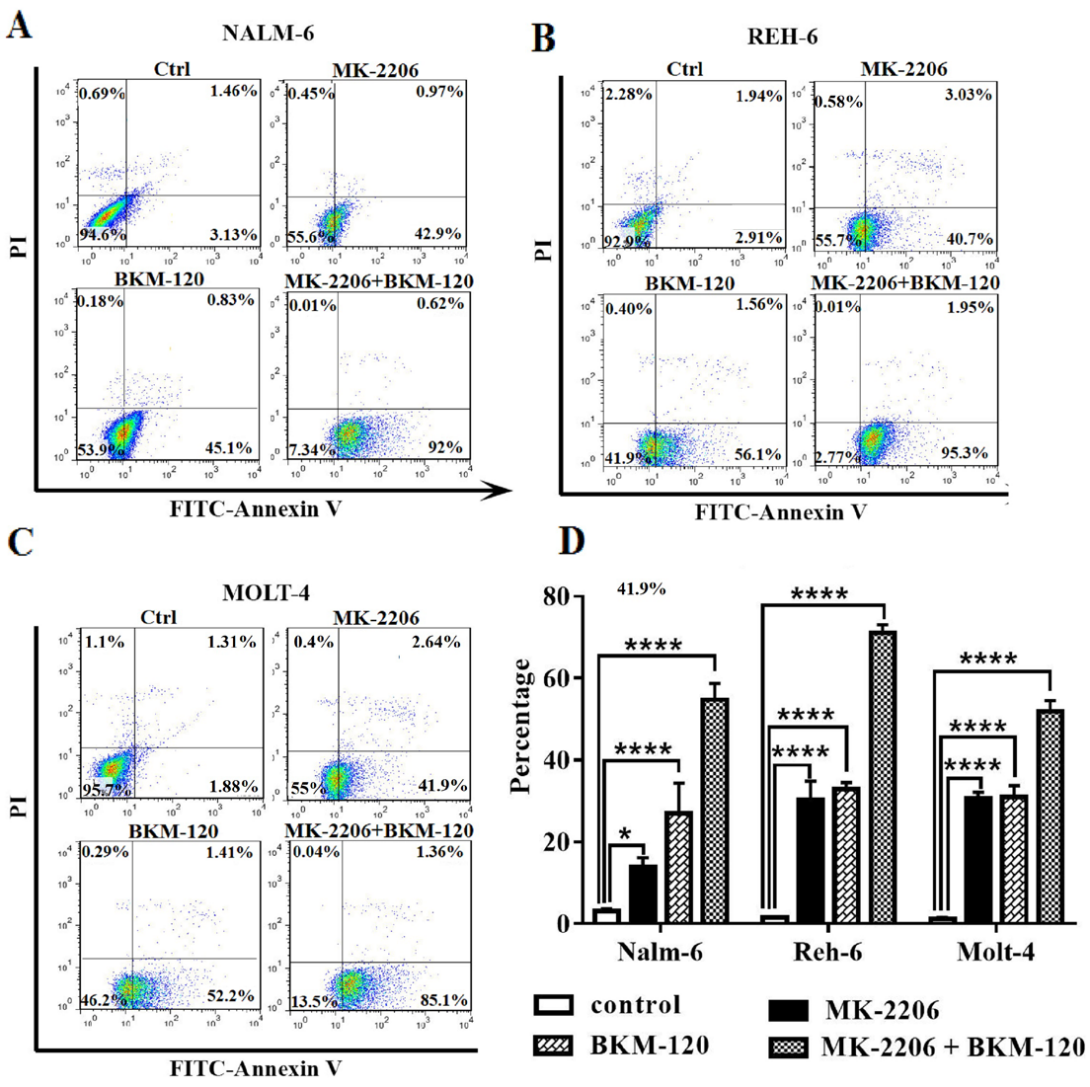
**MK-2206 and BKM-120 induced apoptosis in T and B-cell lines.** Nalm-6 (A), Reh-6 (B) and Molt-4 (C) cell lines were treated for 48 h with IC50 of MK-2206 and BKM-120. Then, flow cytometric analysis of stained cells with Annexin V/PI was performed. The percentage of annexin V–positive fractions were measured and analyzed‏ (D).‏ Data are representative of three independent experiments and values are expressed in mean‏ ± SD. ns: non-significant; * *P* < 0.05; **** P < 0.0001.

Nalm-6, Reh-6 and Molt-4 cell lines were treated with BKM-120 and MK-2206 inhibitors for 48 hours and apoptosis were evaluated by Annexin V/PI staining. Both inhibitors significantly increased the percentage of apoptosis in all cell lines. BKM-120 and MK-2206 had somewhat the same effect on apoptosis in Reh-6 and Molt-4 cell lines. BKM-120 had an intensive effect on apoptosis rather than MK-2206 in Nalm-6 cell line. Interestingly, the percentage of apoptotic positive cells strongly increased when we used both drugs together in all cell lines (Figure 2).

**Figure 3:**
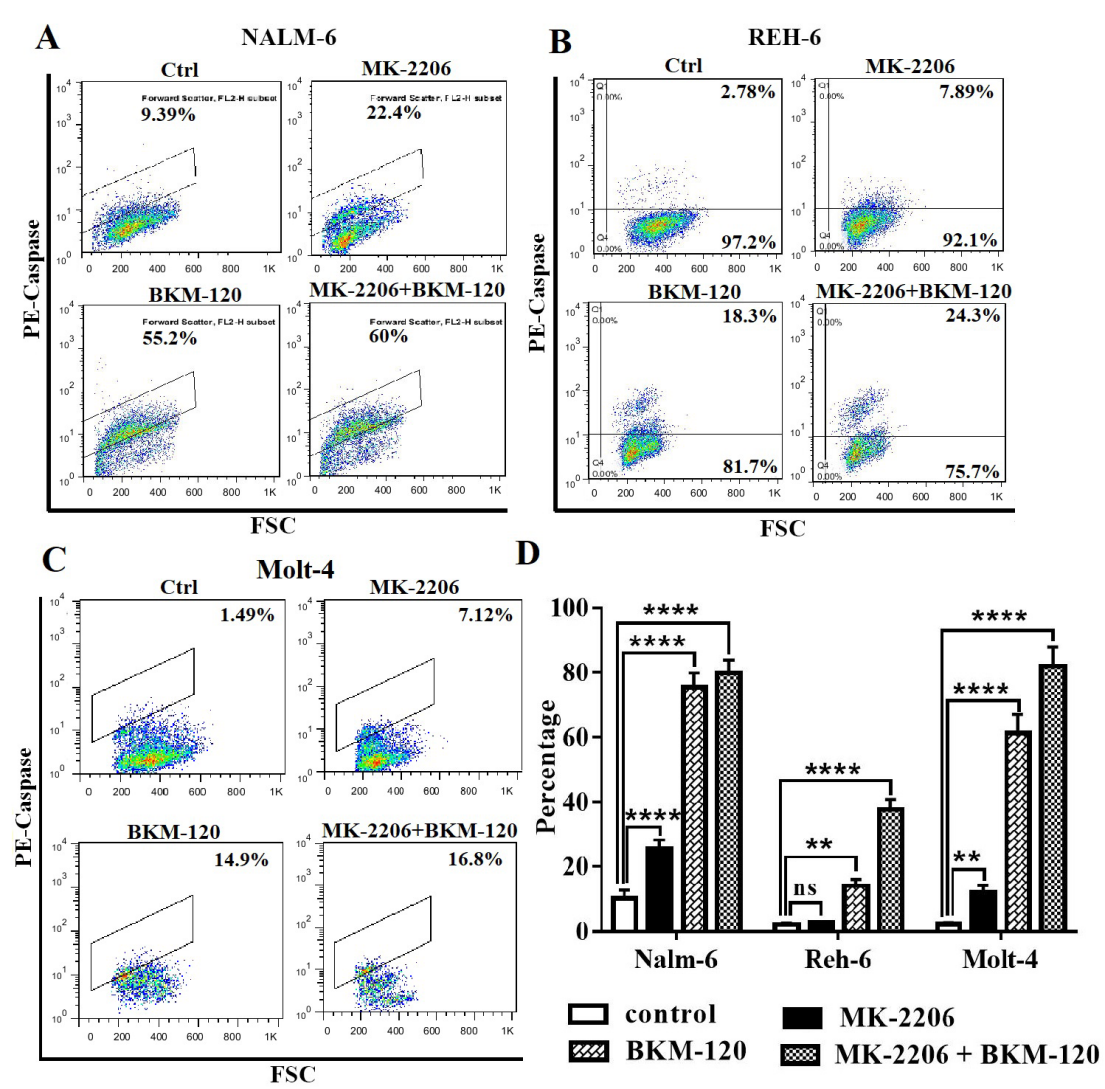
** Flow cytometry plots of caspase3 activation following inhibition of PI3K and Akt in T and B-cell lines.** Nalm-6 (A), Reh-6 (B) and Molt-4 (C) cell lines were treated with IC50 of BKM-120 and MK-2206 for 48 h, afterward stained with caspase3 and flow cytometric analysis was performed. The percentage of caspase3 activated fractions were measured and analyzed (D). Data are representative of three independent experiments and values are expressed in mean‏ ± SD. ns: non-significant; **P< 0.01; **** P < 0.0001.

We also evaluated caspase3 expression to more strengthen this observation, which has a critical role in apoptotic cell death. The apoptotic effect due to activation of caspases-3 significantly increased following treatment with each drug alone in comparison with the control group in all cell lines, although BKM-120 had a greater apoptotic effect relative to MK-2206. Using inhibitors together had a higher rate of apoptosis rather than using alone in all cell lines (Figure 3).

**Figure 4:**
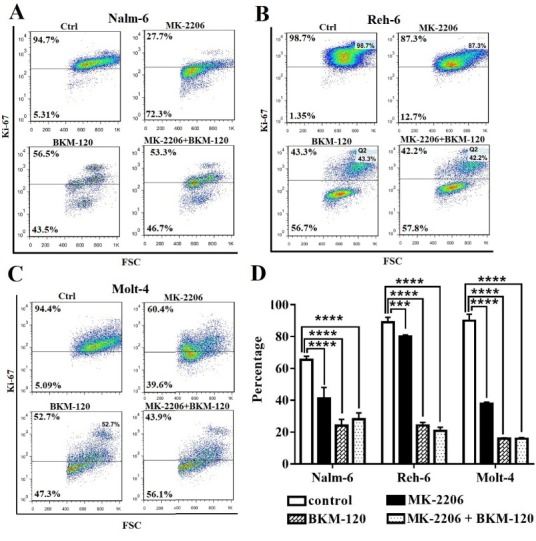
** Flow cytometry plots of Ki67 expression following inhibition of PI3K and Akt in T and B-cell lines.** Nalm-6 (A), Reh-6 (B) and Molt-4 (C) cell lines were treated with IC50 of BKM-120 and MK-2206 for 48 hours then stained with Ki67 and analyzed by flow cytometry. The percentage of Ki67–positive fractions were measured and analyzed (D). Data are representative of three independent experiments and values are expressed in ± SD. ns: non-significant; *** *P*< 0.001;‏ **** P < 0.0001.

To understand whether inhibitors affect proliferation of cell lines, we evaluated Ki67 expression following using these inhibitors. The proliferation of treated groups significantly reduced in comparison with the control group in all cell lines, especially BKM-120 treated groups shown more increased KI67 expression in comparison with MK-2206 inhibitor treated groups in all cell lines. KI67 expression did not notable change when inhibitors administrated together in comparison with BKM-120 alone in all cell lines (Figure 4).

### BKM-120 and MK-2206 efficiently inhibited PI3K/Akt signaling

**Figure 5:**
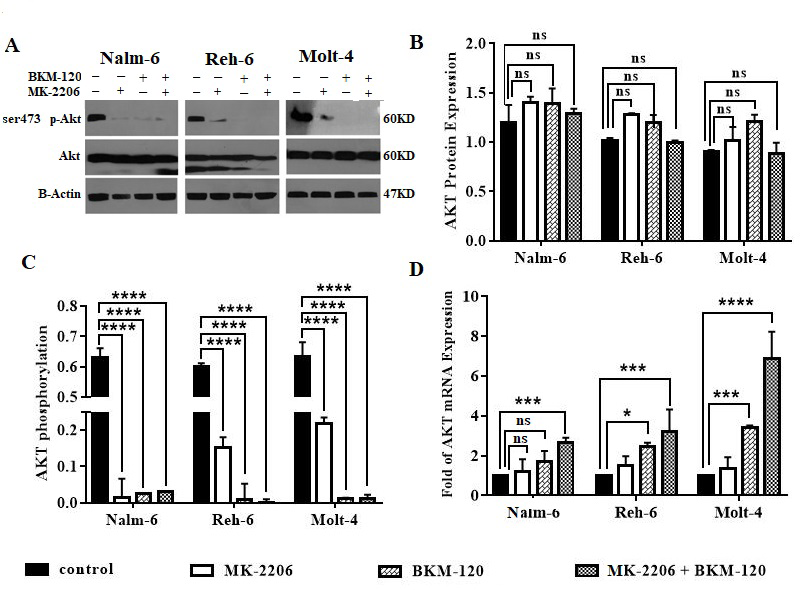
**The mRNA expression, protein and phosphorylation levels of Akt in T and B-cell lines.** Nalm-6, Reh-6 and Molt-4 cell lines were treated with IC50 of BKM-120 and MK-2206 for 48h and then total RNA was isolated and analyzed by real-time RT-PCR. Protein and phosphorylation levels of Akt were evaluated by western blot (A). The expression levels of total Akt bands were quantified by‏ densitometry and normalized to β-actin (B). The expression levels of P-Akt bands were quantified by densitometry and normalized to total Akt (C). The mRNA expression levels of Akt for all cell lines (D). Data are representative of three independent experiments and values are expressed in mean‏ ± SD. ns: non-significant; * *P* < 0.01; *** *P*< 0.001; **** P < 0.0001.

To inspect the impact of PI3K and Akt inhibition on Akt gene and protein expression and phosphorylation, we performed quantitative RT-PCR and western blot analysis after using BKM-120 and MK-2206 inhibitors and a combination of them for 48 hours. As displayed in Figure 5, the mRNA expression level of Akt increased after using inhibitors separately in comparison with the control group in all cell lines. mRNA expression level was higher and significant when cells treated with BKM-120 inhibitor in comparison with MK-2206 treated group. The simultaneous use of inhibitors showed a higher mRNA expression in all cell lines (Figure 5C). The total protein level of Akt there was no significant change after treatment in all cell lines (Figure 5 A and D). Then, evaluation of the phosphorylation status of Akt showed that treated cell lines with BKM-120 and MK-2206 inhibitors and a combination of them resulted in a significant decrease in Ser 473 p-Akt level (Figure 5A and B).

### PI3K and Akt inhibition enhanced PTEN phosphorylation and activity and induced up regulation of PTEN mRNA

**Figure 6:**
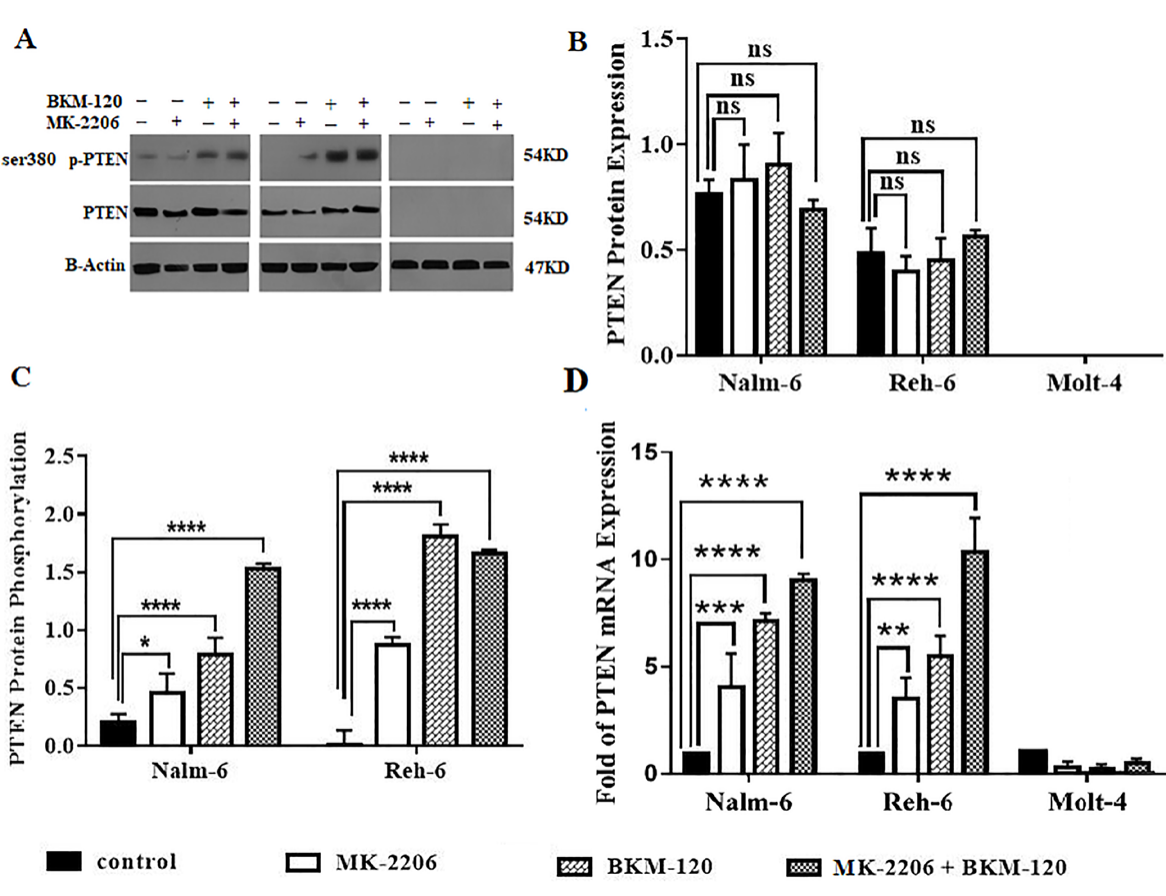
**The mRNA expression, protein and phosphorylation levels of PTEN in T and B-cell lines.** Nalm-6, Reh-6 and Molt-4 cell lines were treated with IC50 of BKM-120 and MK-2206 for 48h and then total RNA was isolated and analyzed by real-time RT-PCR. Protein and phosphorylation levels of PTEN were evaluated by western blot (A). The expression levels of total PTEN bands were quantified by densitometry and normalized to β-actin (B). The expression levels of P-PTEN bands were quantified by densitometry and normalized to total Akt (C). The mRNA expression levels of PTEN for all cell lines (D). Data are representative of three independent experiments and values are expressed in mean ± SD. ns: non-significant; *P< 0.01; *** P < 0.001; **** P < 0.0001.

PTEN as a tumor suppressor negatively controls the activity of PI3K/Akt pathway. Different epigenetic and genetic abnormality, mutations and functional deficiency of PTEN lead to hyperactivity of PI3K/Akt pathway in many of cancer cells.^33^ We evaluated the effect of PI3K and Akt inhibition on the PTEN mRNA and protein expression and phosphorylation. Nalm-6, Reh-6 and Molt-4 cells were treated with IC50 of BKM-120 and MK-2206 inhibitors and a combination of them for 48 hours to measure changes in PTEN gene and protein expression and phosphorylation. We used Real Time PCR assay to assess the effect of PI3K and Akt inhibition on the PTEN mRNA. As shown in (Figure 6C) PTEN gene levels were very low in Molt-4 cell line as expected, because of Molt-4 cell line is PTEN negative. The mRNA relative expression of PTEN significantly increased in BKM-120 and MK-2206 treated groups compared to the control group in Nalm-6 and Reh-6 cell lines. This enhancement is higher when cells treated with BKM-120 inhibitor relative to MK-2206 group. The use of combination inhibitors caused the expression of PTEN gene increased to the highest level in all cell lines. We then performed western blot analysis to evaluate the effect of treatment inhibitors on protein expression alone or in combination with each other. PTEN protein expression levels had no a significant change in the treated group compared to the control group in all cell lines (Figure 6A and D). Inhibition of PI3K and Akt was able to increase PTEN phosphorylation. As observed in (Figure 6A and B) BKM-120 treated group resulted in higher PTEN phosphorylation rather than MK-22-06 treated group. The simultaneous use of inhibitors did not change PTEN phosphorylation in comparison with BKM-120 in Reh-6 cell line, but caused a significant increase in PTEN phosphorylation rather than other groups in Nalm-6 cell line. Taken together, inhibitors intensively increased PTEN phosphorylation in pre B-cell line especially Reh-6 cell line.

### PI3K and Akt inhibition induced up regulation of P53 mRNA and protein

**Figure 7:**
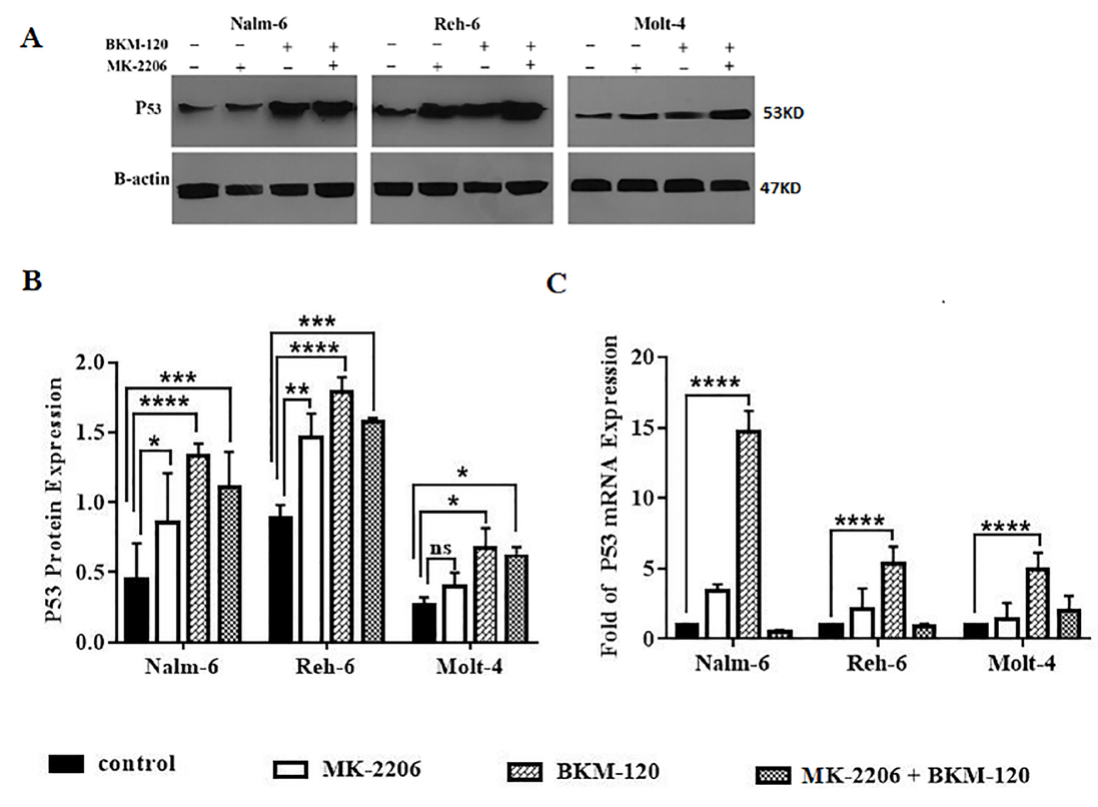
**The mRNA expression and protein levels of P53 in T and B-cell lines.** Nalm-6, Reh-6 and Molt-4 cell lines were treated with IC50 of BKM-120 and MK-2206 for 48h and then mRNA expression and protein levels of PTEN were evaluated. Protein and phosphorylation levels of PTEN were evaluated by western blot (A). The expression levels of total P53 protein bands were quantified by densitometry and normalized to β-actin (B). The mRNA expression levels of P53 for all cell lines (C). Data are representative of three independent experiments and values are expressed in mean ± SD. ns: non-significant; * *P*< 0.05; ** P < 0.01; *** P < 0.001; **** P < 0.0001.

Inhibition of PI3K and Akt caused upregulation of P53 mRNA and protein expression levels when calibrated with the control group in all cell lines. As shown in Figure 7, the mRNA and protein expression levels of P53 increased after treatment with IC50 of BKM-120 and MK-2206 inhibitors and combination of them for 48h in all cell lines. Interestingly, PI3K inhibition by BKM-120 could effectively enhance the expression of P53 mRNA and protein expression relative to Akt inhibition alone or using inhibitors together. BKM-120 treated group had a significant increase in P53 mRNA and protein expression level in all cell lines in comparison with the control group. The enhancement of P53 mRNA affected by BKM-120 especially in PTEN positive Nalm-6 cell line was notable. MK-2206 treated group had a significant increase in P53 protein expression level in Nalm-6 and Reh-6 cell lines in comparison with the control group. An interesting observation is that using the combination of inhibitors resulted in less increasing of P53 mRNA expression level rather than other treated groups in comparison to the control group in all cell lines. Although P53 protein expression level significantly increased compared to control group in all cell lines, this increasing was not more than using BKM-120 inhibitor alone. In PTEN protein positive Nalm-6 and Reh-6 cell line P53 protein levels were more than PTEN protein negative Molt-4 cell line and inhibitors caused more increase of P53 protein levels in comparison to Molt-4 cell line.

### IL-7 rescued PI3K/AKT signaling inhibition

**Figure 8:**
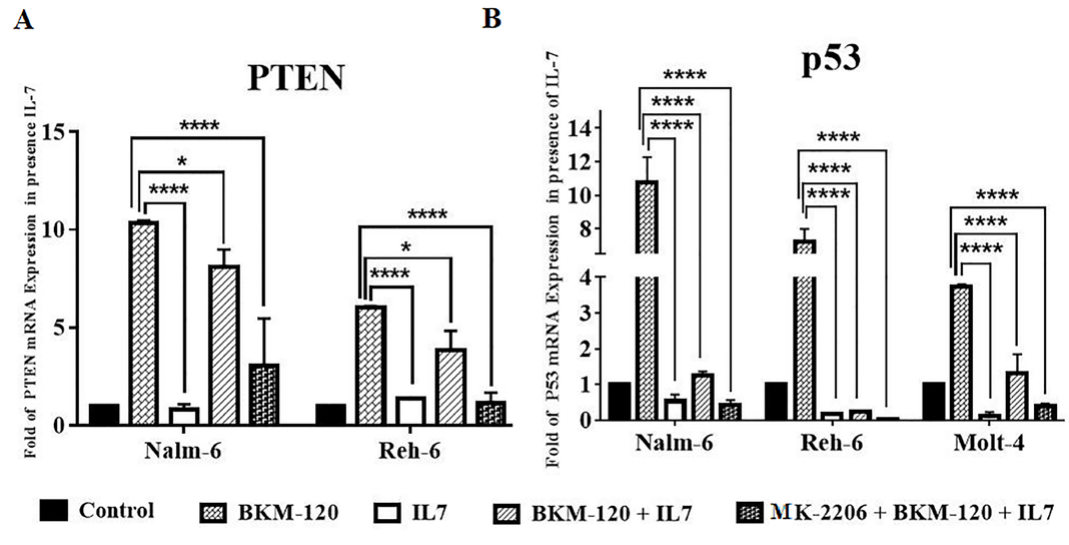
**The mRNA expression levels of PTEN and P53 in T and B-cell lines in presence of IL-7.** Nalm-6, Reh-6 and Molt-4 cell lines were treated with IC50 of BKM-120 and MK-2206 in presence of IL-7 for 48h and then mRNA expression levels of PTEN (A) and P53(B) were evaluated. Data are representative of three independent experiments and values are expressed in mean ± SD. ns: non-significant; * P < 0.05; **P < 0.01; **** P < 0.0001.

Because IL-7 is an environmental cytokine that influences the development of B-cell and T-cell, we investigated effect of IL-7 on PI3K and Akt inhibition. Therefore, effect of PI3K and Akt inhibition on mRNA expression of key agent of PI3K/Akt signaling pathway evaluated in the presence of IL-7 (10 ng/mL of IL-7 that purchased from PeproTech, USA). For this purpose, because BKM-120 had an intensive effect on cell lines proliferation and apoptosis rather than MK-2206, cell lines treated with IC50 of BKM-120 in the presence of IL-7 for 48 hours. Simultaneous treatment of cells with BKM-120 and IL-7 or with MK-2206, BKM-120 and IL-7 showed that inhibition of PI3K by BKM-120 in the presence of IL-7 caused downregulation of mRNA expression level of PTEN and P53 in comparison with using PI3K inhibitor alone (Figure 8 A and B).

## Discussion

The PI3K/Akt signaling pathway dysregulation is effectively involved in cancer onset and progression, making this pathway an interesting target for therapeutic intervention.^34,35^ In this regard, development of new therapeutic strategies against ALL cells for negative modulation of PI3K/Akt signaling is extremely important to achieve better clinical outcomes and restore the functional balance between tumor suppressors and growth signaling pathways.

Vertical inhibition of different targets of the PI3K/Akt signaling leads to better outcomes than use of using single or dual inhibitors in solid tumor treatment.^36-38^ Earlier studies indicated that multiple negative and positive feedback regulatory loops were involved in signal transduction of the PI3K/Akt network.^39^ Therefore, to overcome feedback loops limitation against antitumor effects of PI3K and Akt inhibitors given in monotherapy, simultaneously targeting PI3K/Akt signaling pathway at different levels with different inhibitors might be a promising therapeutic strategy to treat leukemia.

In some previous studies, the usual dual approach in targeting the PI3K/Akt pathway was combining a PI3K inhibitor with a mTOR inhibitor. Interestingly, some rapalogs amplified mTORC2-dependent Akt phosphorylation on Ser 473 and increased cell survival via inhibition of a negative feed-back loop based on mTORC1/p70S6K/IRS1/PI3K.^40,41^ It seems that combination of a PI3K inhibitor with an Akt inhibitor have greater antitumor activity in cancer treatment.

Hence, the present study aims to investigate the therapeutic potential of BKM-120 and MK-2206 for inhibition of PI3K/Akt signaling activity by regarding PTEN and P53 as two main tumor suppressors associated with this pathway. We showed that both inhibitors decreased cell viability, proliferation, and induced apoptosis in PTEN positive Pre B-ALL cell lines (Nalm-6 and Reh-6) and PTEN deleted T-cell line (Molt-4), whereas PTEN positive T-cell line (Dnd-41) was not sensitive to inhibitors. Furthermore, inhibition of PI3K pathway exerted a greater anti-cancer effect than Akt signaling inhibition. BKM-120, the pan-class I PI3K inhibitor, was more powerful than the allosteric Akt inhibitor MK-2206, in both T and Pre B-ALL cell lines. Inhibition with BKM-120 and MK-2206 showed additive efficiency, improved antitumor effectiveness on leukemic cells, enhanced apoptosis, and decreased proliferation in comparison to using each drug alone. Both BKM-120 and MK-2206 inhibited Akt phosphorylation on Ser473, whereas there were no significant changes in total Akt protein levels, confirming the efficacy of the drugs in inhibition of PI3K/Akt signaling activity. However, Akt mRNA increased in all cell lines, unlike the protein levels, which did not significantly change, indicating that Akt was inactivated at the posttranscriptional level.

Similar results were observed in several cancer models. For example, it was previously reported that LY294002 and rapamycin had a much greater degree antiproliferative effect in PTEN-negative breast cancer cells, indicating that PTEN-negative breast cancer cells were more sensitive to PI3K inhibition.^42,43^

Also previous research showed that wortmannin, a PI3K inhibitor, by mimicking PTEN function and enhancing of P53 stability, could induce sensitization to treatment with p53-inducing drugs in refractory tumors with wt p53.^44^ Silva et al demonstrated that LY294002 treatment was not affective against T-ALL patient samples with normal levels of PTEN expression, PTEN phosphorylation, and PI3K/Akt pathway activation, whereas other PTEN-expressing samples showing PI3K/Akt pathway hyperactivation were sensitive to LY294002 treatment. Normal levels of PTEN protein expression in cells were not necessarily synonymous with normal function of PTEN. PI3K/Akt pathway hyperactivation occurs as a result of posttranslational modification of PTEN by CK2-mediated phosphorylation and ROS-dependent oxidation.^45^

We showed that Dnd-41 cell line with PTEN positive status did not show sensitivity to the inhibitors, but Molt-4 was sensitive to PI3K and Akt inhibitors. On the other hand, Nalm-6 and Reh-6 cell lines with PTEN positive status were sensitive to PI3K and Akt inhibitors that may be implies to posttranslational modification of PTEN and loss of normal function of PTEN in these cell line. Furthermore, Earlier studies indicated that Akt was emphatically activated in PTEN-negative cell lines. Activation of Akt results in Mdm2 phosphorylation and promotes translocation of Mdm2 from the cytoplasm to the nucleus. Moreover, inhibition of PI3k/Akt by PTEN results in accumulation of MDM2 in the cytoplasm. In PTEN-positive cells, MDM2 is localized mainly in the cytoplasm, whereas MDM2 is expressed mostly in the nucleus in PTEN-negative cells. However, it showed that PTEN-expressing cell lines did not display significant reduction of PI3k and Akt-p. In addition, transfection of PTEN did not significantly inhibit Akt-p expression in PTEN negative cell lines. These results indicate that PTEN-mediated cell survival and apoptosis are partially independent of the PI3k/Akt pathway.^46^

However, assessment of PI3K/Akt pathway activity in patient samples at diagnosis could be helpful to determine treatment programs.

PTEN as an important tumor suppressor that negatively controls PI3K/Akt pathway through dephosphorylation of PIP3.^33^ Various genetic and epigenetic alterations, as well as translational and post-translational modifications of PTEN imply decreased or complete loss of PTEN protein expression and activity in various cancers.^47^ Another important tumor suppressor is p53 that acts as a transcription factor and is involved in activation of the gene network responsible for a cell cycle arrest and apoptosis in response to DNA damages and cell stresses.^48,49^ Maintenance of low levels of p53 is required for cell viability in normal cells that is provided by its rapid ubiquitination. Stabilization of p53 is essential for its tumor suppressor function.^50^ Activation of PI3K/Akt signaling negatively controls p53 levels through promotion of Mdm2 movement in the nucleus.^51^ Mdm2 is an oncoprotein regulating the p53 protein levels and transcriptional activation through ubiquitin ligase activity.^22^ PTEN inhibits activation of PI3K/Akt signaling, prevents translocation of Mdm2 in the nucleus and positively regulates p53. Thus, it is clear that PTEN can control P53 function via inhibition of PI3K/Akt signaling with decrease of Mdm2 and p53 interaction and degradation.^51^ There are multiple layers of crosstalk between PTEN and P53,^44,52^ including transcription and protein levels affecting the cell death and survival.^53^ Mutation and inactivation of these genes were the most common elements in human cancers.^54^ Moreover, lack of PTEN tumor suppressor function cooperates with p53 loss for tumor development.^23^ Expression of PTEN in PTEN-null glioblastoma cells increases the expression of p53 target genes involved in cell cycle arrest.^55^ In fact, p53 upregulates PTEN, and PTEN protects p53 from survival signals by antagonizing PI3K/Akt signaling. PTEN–p53 associations demonstrated their dependence on each other in tumor suppression activity.^56^ We illustrated inhibition of PI3K/Akt signaling with BKM-120 and MK-2206 inhibitors upregulated PTEN gene and increased PTEN protein phosphorylation, whereas total PTEN protein levels did not change significantly. In addition, we found that inhibition of PI3K and Akt by BKM-120, and MK-2206 inhibitors caused upregulation of P53 mRNA and protein expression levels in all cell lines. It is likely that enhanced levels of PTEN phosphorylation upon PI3K**/**AKT inhibition affect p53 stability and increase P53 protein levels in all cell lines.

These findings are consistent with previous observations that mutation and loss of PTEN lead to deficiency or absence of p53 protein. Our results showed that p53 protein in PTEN negative cell line (Molt-4) displayed lower levels than PTEN positive cell lines.

IL-7 cytokine, as one of the important growth factors produced by bone marrow and thymic stroma, functions as a powerful proliferative stimulus for leukemia cells.^57,58^

IL-7 regulates lymphoid development and may stimulate proliferation and survival of ALL cells.^59^ IL-7 as an antiapoptotic factor plays a crucial role in normal T cell development through down-regulating the expression of the cyclin dependent kinase (cdk) inhibitor p27kip1, resulting in up-regulation of Bcl-2 expression, and cell cycle progression.^60^ Severe T cell deficiency was observed in defective IL-7R expression.^61^ IL-7 stimulates proliferation of pro–B cells and development of early B-cell stages.^62^ IL-7-mediated signaling contributes to survival and cell cycle progression^63^ and it has been demonstrated that IL-7 can active PI3K/AKT signaling in T-ALL.^18^ A previous study indicated that LY294002, a PI3K inhibitor, could prevent induction of proliferation by IL-7 in T ALL cells.^64^ Another study also showed that inhibition effect of rapamycin in B-precursor acute lymphoblastic leukemia lines could be reversed by IL-7. Our finding showed that inhibitory effects of BKM-120 and MK-2206 in the presence of IL-7 could be reversed, and IL-7 was capable of reversing the inhibitory effect of inhibitors by downregulation of PTEN and p53 mRNA levels.^65^

## Ethical Issues

This study was approved by ethical committee of Tabriz University of Medical Sciences with ethical number: TBZMED.REC.1394.752.

## Conflict of Interest

Authors declare no conflict of interest in this study.

## Acknowledgments

This work was supported by Tabriz University of Medical sciences under Grant (5/104/996). We thank the Stem Cell Research Center for technical support.
